# Case Report: Bilateral adrenal hemorrhage and hemophagocytosis in the reticuloendothelial system caused by *Escherichia coli* in a young woman: an autopsy case

**DOI:** 10.3389/fmed.2025.1496961

**Published:** 2025-03-11

**Authors:** Yudai Tanaka, Koji Hayashi, Ei Kawahara, Shin-Ichiro Azuma, Maho Hayashi, Yuka Nakaya, Asuka Suzuki, Midori Ueda, Rei Asano, Hiromi Hayashi, Toyoaki Miura, Kouji Hayashi, Mamiko Sato, Yasutaka Kobayashi

**Affiliations:** ^1^Department of Rehabilitation Medicine, Fukui General Hospital, Fukui, Japan; ^2^Department of Infectious Disease, Faculty of Medical Science, University of Fukui, Fukui, Japan; ^3^Department of Pathology, Fukui General Hospital, Fukui, Japan; ^4^Department of Internal Medicine, Fukui General Hospital, Fukui, Japan; ^5^Graduate School of Health Sciences, Fukui Health Science University, Fukui, Japan

**Keywords:** bilateral adrenal hemorrhage, hemophagocytic lymphohistiocytosis, disseminated intravascular coagulopathy, sepsis, *Escherichia coli*

## Abstract

**Background:**

Sepsis can lead to life-threatening complications such as hemophagocytic lymphohistiocytosis (HLH) and bilateral adrenal hemorrhage (BAH), commonly known as Waterhouse-Friderichsen syndrome. HLH involves severe inflammation and organ damage, often linked with infections and autoimmune diseases, while BAH leads to adrenal insufficiency, typically caused by *Neisseria meningitidis*, though *Escherichia coli* (*E. coli*) is a rare cause.

**Case presentation:**

A 27-year-old Japanese woman, with a history of diabetes mellitus and obesity, presented with a complicated urinary tract infection. She commenced oral levofloxacin treatment five days before hospital admission. Her medications included prednisolone 5 mg/day, trimethoprim-sulfamethoxazole for pyoderma gangrenosum, and oral hypoglycemic agents. Upon admission, her vital signs, including body temperature and blood pressure, were stable, and her body mass index was recorded at 49.8. Laboratory tests indicated elevated white blood cell count and C-reactive protein. She was diagnosed with a urinary tract infection and was treated with levofloxacin and trimethoprim-sulfamethoxazole. On day 16 of hospitalization, her general condition rapidly worsened, leading to her sudden death on day 18. Premortem blood tests showed cytopenia, and blood cultures tested positive for *E. coli*. Autopsy imaging using computed tomography revealed enlargement and high density of the left adrenal gland. Postmortem pathology identified bilateral adrenal hemorrhages with focal necrosis. Extensive erythrophagocytic macrophages were observed in the bone marrow, spleen, and liver. Disseminated intravascular coagulopathy was confirmed through the detection of a renal glomerular fibrin thrombus.

**Conclusion:**

We concluded that *E. coli* infection had caused multiple fatal conditions, such as disseminated intravascular coagulopathy, bilateral adrenal hemorrhage, and suspected hemophagocytic lymphohistiocytosis leading to the death of the young woman.

## Introduction

Sepsis, recognized as a life-threatening organ dysfunction, arises from a dysregulated host response to infection (1). This condition can lead to a range of severe complications, one notable example being hemophagocytic lymphohistiocytosis (HLH). HLH is a fatal inflammatory syndrome that is pathologically characterized by extensive hemophagocytic activity by macrophages in various organs (2–4). While HLH in children predominantly occurs as a familial disorder, adult-onset HLH is typically associated with various underlying conditions such as viral infections, lymphoproliferative diseases, and autoimmune disorders (5, 6). HLH caused by bacterial infection is less common (5, 6), and HLH secondary to *Escherichia coli* (*E. coli*) infection is rarely reported (7, 8).

Bilateral adrenal hemorrhage (BAH), classically known as Waterhouse-Friderichsen syndrome, was initially described by Rupert Waterhouse in 1911 and Carl Friderichsen in 1918 (9). BAH is a rare syndrome associated with adrenal insufficiency that can occasionally become life-threatening. It is linked to a spectrum of infectious and non-infectious conditions (9, 10). The most commonly identified infectious agent is *Neisseria meningitidis* (10), as initially noted by Friderichsen (9). Nonetheless, a variety of bacteria have been implicated in the etiology of this syndrome (9), with *E. coli*-related instances being notably rare (11, 12).

In the present case, we report that the postmortem pathological examinations show the unusual co-occurrence of BAH and hemophagocytosis in the reticuloendothelial system as complications in the progression of sepsis, instigated by an infection with the widely encountered bacterium, *E. coli*.

## Case description

A 27-year-old Japanese woman, with a history of diabetes mellitus and obesity, and previous diagnosis of a complicated urinary tract infection (UTI) based on an elevated serum inflammatory response and bacteriuria, was prescribed oral levofloxacin, which had been initiated 5 days prior to admission. Her medical history included long-term treatment with prednisolone, trimethoprim-sulfamethoxazole for pyoderma gangrenosum and oral hypoglycemic agents such as biguanides, SGLT2 inhibitors, and GLP-1 receptor agonists for latent autoimmune diabetes started 8 years ago. Pyoderma gangrenosum developed one year before admission, and she was started on 50 mg/day of prednisolone, which was tapered to 5 mg/day to account for the effect on her underlying diabetes mellitus, while she had recurrent refractory leg ulcers and skin and soft tissue infections that were repeatedly treated with antibacterial agents. Five days before her admission, blood tests revealed HbA1c level of 6.9%, C-peptide immunoreactivity (CPR) level of 7.2 ng/mL, triglycerides (TG) of 192 mg/dL, HDL cholesterol of 66 mg/dL, and LDL cholesterol of 137 mg/dL. Three days before her admission, she developed anorexia and headache. Upon admission, her vital signs, including body temperature and blood pressure, were within normal ranges, while her body mass index was recorded at 49.8. There were no abnormal findings in the chest or abdomen, and the neurological evaluation was unremarkable. Laboratory investigations revealed an elevated white blood cell count and increased CRP levels (Table 1). Urinary analysis showed the presence of ketones and bacteria; however, pyuria was not detected (Table 1). Her venous blood gas analysis showed a pH of 7.360 and HCO3- of 20.5. Although the urinary analysis results were atypical, she was diagnosed with a complicated UTI based on her clinical history of recurrent UTIs and treated with levofloxacin and trimethoprim-sulfamethoxazole. In addition, prednisolone was dosed up to 6 mg per day. She was not eating enough and was admitted to the hospital for intravenous therapy and blood glucose monitoring. Despite the ongoing treatment and an initial improvement in her symptoms during the course of hospitalization, her condition deteriorated rapidly on day 16 of hospitalization, leading to her sudden death on day 18. She presented with nausea and vomiting, with complaints of diarrhea and decreased urinary output before her death. Premortem blood tests indicated bicytopenia (Table 2), and two sets of blood cultures yielded positive results for *E. coli* using the BD BACTEC™ bottle.

**TABLE 1:** Laboratory findings of blood and urine tests on admission.

Inspection items	Result	Reference range	Inspection items	Result	Reference range
White blood cell count	12,400/μl	(3,300–8,600)	Creatinine	0.54 mg/dl	(0.46–0.79)
Red blood cell count	428 × 10^4^ /μl	(386–492 × 10^4^)	Sodium	143 mmol/l	(138–145)
Hemoglobin	11.2 g/dl	(11.6–16.0)	Potassium	4.1 mmol/l	(3.6–4.8)
Hematocrit	36.3%	(35.1–44.4)	Chloride	107 mmol/l	(101–108)
Blood platelet	41.2 × 10^4^ /μl	(15.8–34.8)	Glucose	101 mg/dl	(73–109)
Total protein	8.1 g/dl	(6.6–8.1)	C-reactive protein	2.25 mg/dl	(0.00–0.14)
Albumin	4.4 g/dl	(4.1–5.1)	Urinary analysis		
Alkaline phosphatase	93 U/l	(38–113)	White blood cell	–	
Aspartate aminotransferase	117 U/l	(13–30)	Urobilinogen	–	
Alanine aminotransferase	106 U/l	(7–30)	Protein	–	
Lactate dehydrogenase	138 U/l	(124–222)	Occult blood	+	
Creatine kinase	526 U/l	(41–153)	Keton body	+++	
Amylase	33 U/l	(44–132)	Sugar	+	
Blood urea nitrogen	10.7 mg/dl	(8.0–20.0)	Bacteria	+	

**TABLE 2:** The results of blood test and blood gas analysis immediately after death.

Inspection items	Result	Reference range	Inspection items	Result	Reference range
White blood cell count	23,200/μl	(3,300–8,600)	Blood urea nitrogen	35.7 mg/dl	(8.0–20.0)
Red blood cell count	381 × 10^4^ /μl	(386–492 × 10^4^)	Creatinine	3.99 mg/dl	(0.46–0.79)
Hemoglobin	10.1 g/dl	(11.6–16.0)	Sodium	148 mmol/l	(138–145)
Hematocrit	35.0%	(35.1–44.4)	Potassium	5.4 mmol/l	(3.6–4.8)
Blood platelet	2.1 × 10^4^ /μl	(15.8–34.8)	Chloride	102 mmol/l	(101–108)
Total protein	5.4 g/dl	(6.6–8.1)	Glucose	101 mg/dl	(73–109)
Albumin	2.6 g/dl	(4.1–5.1)	C-reactive protein	> 30.0 mg/dl	(0.00–0.14)
Alkaline phosphatase	335 U/l	(38–113)			
Aspartate aminotransferase	57 U/l	(13–30)	Blood gas analysis		
Alanine aminotransferase	49 U/l	(7–30)	pH	6.66	(7.38–7.46)
Lactate dehydrogenase	428 U/l	(124–222)	pO2	46.0	(74.0–108.0)
Creatine kinase	75 U/l	(41–153)	pCO2	78.6	(32.0–46.0)
Amylase	15 U/l	(44–132)	HCO3–	8.8	(21.0–29.0)
Gamma-glutamyl transpeptidase	114 U/l	(9–32)	Base excess	−26.1	(−2.0 to 2.0)
Cholinesterase	252 U/l	(201–421)	Standard bicarbonate concentration	5.9	(21.0–29.0)

**FIGURE 1:**
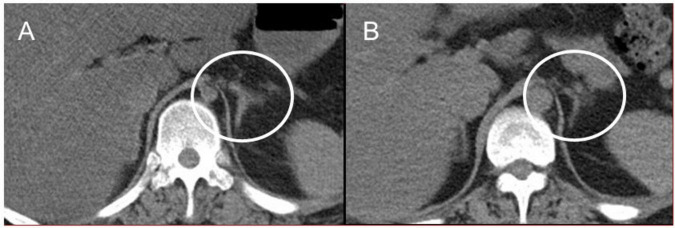
Abdominal CT images. **(A)** Postmortem CT imaging reveals hyperdensity in the left adrenal gland (indicated by a circle), suggestive of adrenal hemorrhage. **(B)** A CT scan performed 5 months prior to admission shows no abnormalities in the left adrenal gland (indicated by a circle). CT, computed tomography.

**FIGURE 2:**
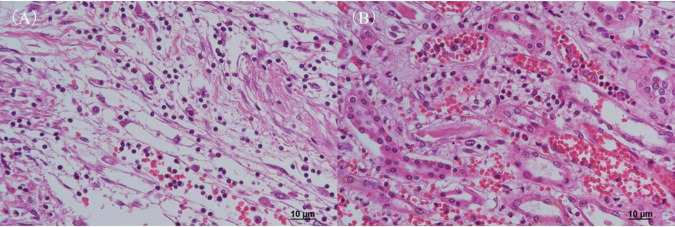
Histopathological examination of the kidney with H&E staining. **(A)** Renal pelvis and **(B)** renal medulla. Both regions show infiltration of inflammatory cells, predominantly neutrophils, along with evidence of hemorrhage, consistent with pyelonephritis. H&E, hematoxylin and eosin.

**FIGURE 3:**
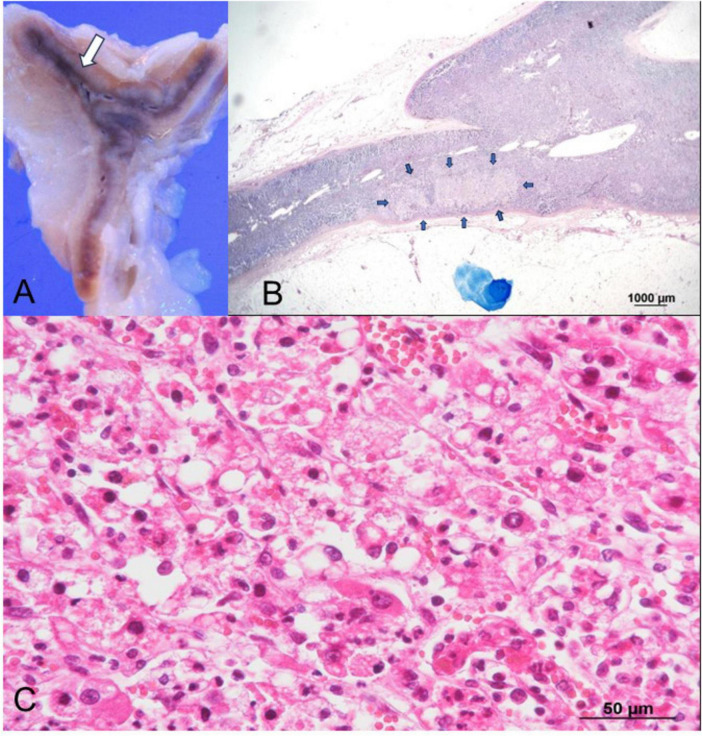
Histopathological findings of the left adrenal gland. **(A)** Gross examination reveals hemorrhage (arrow) in the left adrenal gland. **(B)** Low-magnification Giemsa staining highlights a weakly stained area (arrows) consistent with necrosis, with no bacteria observed. **(C)** Higher magnification of the weakly stained area in **(B)**, examined under H&E staining, reveals extravascular erythrocytes and necrotic cells surrounded by neutrophils, indicating extensive hemorrhages and necrosis. H&E, hematoxylin and eosin.

**FIGURE 4:**
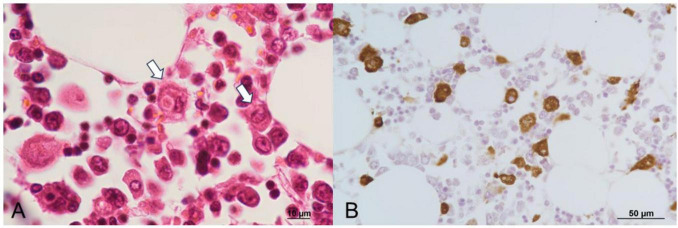
Histopathological findings of the bone marrow. **(A)** Macrophages engulfing erythrocytes, observed with H&E staining. **(B)** Erythrophagocytic macrophages demonstrate CD68 positivity on immunoperoxidase staining. H&E, hematoxylin and eosin.

**FIGURE 5:**
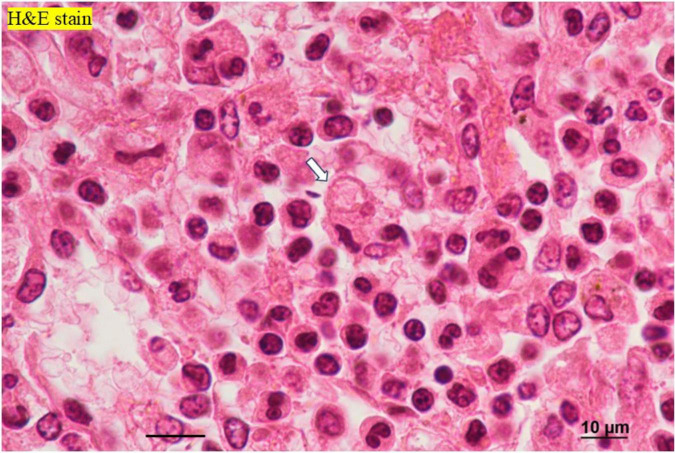
Histopathological findings of the spleen. H&E staining reveals neutrophils (black arrows) and erythrophagocytosis by macrophages (white arrow). H&E, hematoxylin and eosin.

**FIGURE 6:**
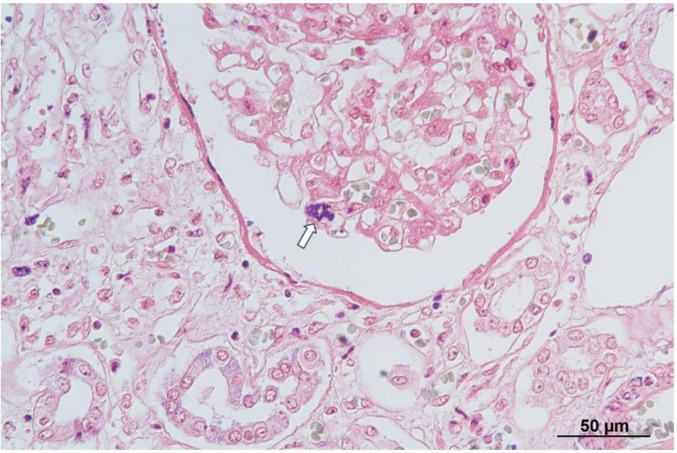
Histopathological findings of the kidney. Phosphotungstic acid-hematoxylin staining reveals a fibrin thrombus (arrow) within the glomerular capillary, indicative of disseminated intravascular coagulopathy (DIC).

Autopsy imaging using computed tomography (CT) showed enlargement and high density of the left adrenal glands compared to its normal appearance on images taken 5 months prior (Figure 1). Postmortem anatomical examinations revealed the presence of an accumulation of inflammatory cells in the renal pelvis and renal medulla. Despite the absence of bacteriological evidence, the pathology report indicated the presence of pyelonephritis (Figure 2). They also revealed an adrenal hemorrhage and necrosis (Figure 3), with the left adrenal gland showing more severe hemorrhage and necrosis compared to the right. Numerous erythrophagocytic macrophages were observed in the bone marrow (Figure 4), spleen (Figure 5), and liver. Acute splenitis with neutrophil infiltration, indicative of sepsis, was also observed in the spleen (Figure 5). The renal glomerular fibrin thrombus, confirming disseminated intravascular coagulopathy (DIC), was identified (Figure 6). Hemorrhagic diathesis secondary to DIC was evident with intra-alveolar, subpleural, subperitoneal, and ileal mucosal bleeding. The likely source of the *E. coli* leading to sepsis was identified as pyelonephritis. The autopsy concluded that the cause of death was acute adrenal insufficiency due to BAH and hemophagocytosis associated with sepsis caused by *E. coli*.

## Discussion

This case report details the autopsy findings of a young woman who succumbed to both BAH and suspected HLH secondary to an *E. coli* infection. HLH is characterized by a wide range of symptoms including fever, organomegaly, hepatomegaly, splenomegaly, lymphadenopathy, liver injury, consumptive coagulopathy, hypertriglyceridemia, pancytopenia, neurologic dysfunction, dermatologic abnormalities, and increased acute-phase reactants, especially serum ferritin (2). Due to the variability of these symptoms, HLH diagnosis can be challenging. According to the HLH-2004 diagnostic guidelines, primarily developed for pediatric cases, clinical diagnosis of HLH should fulfill five out of the following eight criteria: fever, splenomegaly, cytopenias (affecting at least two of three lineages), hypertriglyceridemia and/or hypofibrinogenemia, hemophagocytosis in bone marrow, spleen, or lymph nodes, ferritin ≥ 500 ng/mL, low or absent NK-cell activity, soluble CD25 > 2,400 U/mL (13). However, these criteria have not been validated for secondary HLH (14), making its diagnosis during sepsis particularly difficult. In cases of infection, organomegaly, cytopenia, and elevated serum ferritin levels are indicative of HLH, as they reflect erythrophagocytosis in the lymphoreticular system. In our case, we were unable to test for triglyceride, fibrinogen, ferritin, NK cell activity, or soluble CD25 because the patient died suddenly, and we did not suspect HLH in the premortem phase. In addition, hepatosplenomegaly was not discernible due to significant obesity and concurrent fatty liver. Although a definitive diagnosis of HLH could not be made due to a lack of testing, we actively suspected the possibility of HLH because blood test data showed bicytopenia, pathological findings indicated signs of hemophagocytosis, and there have been reports of HLH in patients with bacterial infections, including *E. coli* (5–8).

Suspecting acute adrenal insufficiency, including classical Waterhouse-Friderichsen syndrome, but constrained by the rapid clinical progression, we could not perform some tests including endocrinological tests. Autopsy findings revealed an enlarged left adrenal gland and bilateral hemorrhage with necrosis of the adrenal cortices. Over the years, there have been reports of BAH related to multiple etiologies, including various systemic bacterial and viral infections (9, 10), with *Neisseria meningitidis* as a notable causative agent of BAH. However, cases of BAH resulting from *E. coli* infection are rare (11–13). Although *E. coli* is a common microorganism, many internists may overlook the complications associated with BAH. BAH, leading to adrenal insufficiency (15, 16), is often predisposed by DIC (17, 18). While antemortem diagnosis of BAH is challenging, serial CT imaging of the abdomen is valuable (15, 16), as confirmed by our autopsy imaging.

As far as we know, there is no previous report regarding the combination of BAH and HLH. However, both conditions are fatal and can be caused by severe infections, including sepsis. Additionally, radiological diagnosis has limitations for both conditions, and several tests are often required, including genetic and pathological tests. In fact, in our case, the diagnosis was made after evidence of these diseases was collected through a postmortem pathological autopsy. Based on these findings, we speculated that the lack of previous reports may be due to underdiagnosis.

The patient had multiple risk factors including diabetes mellitus, severe obesity, and chronic oral corticosteroid use. Although we could not determine her immune status before death, it was likely insufficient due to her history of recurrent infections, including skin and soft tissue infections. In addition, although we did not diagnose pyelonephritis during her pre-mortem phase, we observed evidence of it during the postmortem autopsy. The rapid progression of the infection leading to her death may have been due to her immunocompromised status. The sepsis, secondary to *E. coli* infection from pyelonephritis, likely increased her body’s demand for steroids, and persistent infection-related hypercytokinemia may have ensued. We concluded that the *E. coli* infection precipitated multiple fatal conditions such as DIC, BAH and conditions in which HLH may be suspected, culminating in the young woman’s death.

This study has some limitations: the course of events leading up to her death was so rapid that there were only limited test results available. Specifically, the complications of HLH and BAH were not recognized during her life, and the necessary tests for diagnosing HLH, including hypercytokinemia, were not conducted, which prevented a definitive diagnosis. Additionally, the possibility of familial HLH could not be ruled out. Although fibrin thrombi in the renal glomeruli demonstrated pathomorphological findings consistent with DIC, coagulability studies to confirm DIC could not be performed. Antimicrobial susceptibility testing of the *E. coli* identified postmortem was not performed, so the effectiveness of the levofloxacin and trimethoprim-sulfamethoxazole treatments administered during her illness could not be evaluated. In addition, urine culture was not performed at the time of death, and bacteriological *E. coli* urinary tract infection could not be proven. However, because we found evidence of adrenal hemorrhage, pyelonephritis, and hemophagocytosis in the reticuloendothelial system at autopsy, we believe this is an important case report, despite the limitations mentioned above.

## Conclusion

We presented an autopsy case of a young woman with bilateral adrenal hemorrhage (BAH) and hemophagocytosis in the reticuloendothelial system associated with *Escherichia coli* infection. Although *E. coli* is a common bacterium, it can cause serious complications such as BAH and hemophagocytosis, even in young adults, particularly when the patient is immunocompromised due to steroid therapy or other conditions, potentially leading to death. To our knowledge, there have been no previous reports of simultaneous adrenal hemorrhage and hemophagocytosis induced by *E. coli*, making this a valuable case report.

## Data Availability

The original contributions presented in this study are included in this article/supplementary material, further inquiries can be directed to the corresponding author.

## References

[1] Singer M Deutschman C Seymour C Shankar-Hari M Annane D Bauer M The third international consensus definitions for sepsis and septic shock (Sepsis-3). *JAMA.* (2016) 315:801–10. 10.1001/jama.2016.0287 26903338 PMC4968574

[2] Al-Samkari H Berliner N. Hemophagocytic lymphohistiocytosis. *Annu Rev Pathol.* (2018) 13:27–49. 10.1146/annurev-pathol-020117-043625 28934563

[3] Janka G. Hemophagocytic syndromes. *Blood Rev.* (2007) 21:245–53. 10.1016/j.blre.2007.05.001 17590250

[4] Fisman D. Hemophagocytic syndromes and infection. *Emerg Infect Dis.* (2000) 6:601–8. 10.3201/eid0606.000608 11076718 PMC2640913

[5] Oda Y Urushidani Y Ooi S Endoh A Nakamura R Adachi K Hemophagocytic lymphohistiocytosis in a rheumatoid arthritis patient treated with infliximab. *Intern Med.* (2012) 51:655–7. 10.2169/internalmedicine.51.5687 22449679

[6] Ishii E Ohga S Imashuku S Yasukawa M Tsuda H Miura I Nationwide survey of hemophagocytic lymphohistiocytosis in Japan. *Int J Hematol.* (2007) 86:58–65. 10.1532/IJH97.07012 17675268

[7] Chang K Tang L Lyu R. Spontaneous *Escherichia coli* meningitis associated with hemophagocytic lymphohistiocytosis. *J Formos Med Assoc.* (2006) 105:756–9. 10.1016/S0929-6646(09)60204-7 16959624

[8] Risdall R Brunning R Hernandez J Gordon D. Bacteria-associated hemophagocytic syndrome. *Cancer.* (1984) 54:2968–72. 10.1002/1097-0142(19841215)54:12 6498770

[9] D’Agati M Major B Marangoni M. The waterhouse-friderichsen syndrome. *N Engl J Med.* (1945) 232:1–7. 10.1056/NEJM194501042320101

[10] Guarner J Paddock C Bartlett J Zaki S. Adrenal gland hemorrhage in patients with fatal bacterial infections. *Mod Pathol.* (2008) 21:1113–20. 10.1038/modpathol.2008.98 18500257

[11] Khwaja J. Bilateral adrenal hemorrhage in the background of *Escherichia coli* sepsis: A case report. *J Med Case Rep.* (2017) 11:72. 10.1186/s13256-017-1236-0 28302165 PMC5356297

[12] Yokoyama S Sekioka A Utsunomiya H Hara S Takahashi T Yoshida A. Adrenal abscess as a complication of *Escherichia coli* sepsis in neonates: A case report. *J Pediatr Surg Case Rep.* (2013) 1:328–30. 10.1016/j.epsc.2013.09.001

[13] Henter J Horne A Aricó M Egeler R Filipovich A Imashuku S HLH-2004: diagnostic and therapeutic guidelines for hemophagocytic lymphohistiocytosis. *Pediatr Blood Cancer.* (2007) 48:124–31. 10.1002/pbc.21039 16937360

[14] Otrock Z Daver N Kantarjian H Eby C. Diagnostic challenges of hemophagocytic lymphohistiocytosis. *Clin Lymphoma Myeloma Leuk.* (2017) 17S:S105–10. 10.1016/j.clml.2017.02.017 28760295

[15] Siu S Kitzman D Sheedy P Northcutt R. Adrenal insufficiency from bilateral adrenal hemorrhage. *Mayo Clin Proc.* (1990) 65:664–70. 10.1016/S0025-6196(12)65129-5 2161483

[16] Vella A Nippoldt T Morris J. Adrenal hemorrhage: A 25-year experience at the Mayo clinic. *Mayo Clin Proc.* (2001) 76:161–8. 10.4065/76.2.161 11213304

[17] Mutlu M Karagüzel G Aslan Y Cansu A Ökten A. Adrenal hemorrhage in newborns: A retrospective study. *World J Pediatr.* (2011) 7:355–7. 10.1007/s12519-011-0259-7 21874621

[18] Hori K Yasoshima H Yamada A Sakurai K Ohkubo E Kubota A Adrenal hemorrhage associated with Klebsiella oxytoca bacteremia. *Intern Med.* (1998) 37:990–4. 10.2169/internalmedicine.37.990 9868968

